# Proteomic insights into aprotinin’s immunomodulatory effects in attenuating renal fibrosis in a unilateral ureteral obstruction model

**DOI:** 10.3389/fimmu.2026.1751652

**Published:** 2026-05-01

**Authors:** Xinyu Wang, Ying Shen, Danlei Chen, Lele Ding, Yuanlong Ding, Peilin Gan, Qian Long, Lu Song, Yaling Li, Xin Tian, Jun Zhang, Jian Xu, Yutaka Kakizoe, Shu Yang, Fucheng Luo, Qinyuan Deng

**Affiliations:** 1 Department of Nephrology, Yunnan Provincial Clinical Medical Center for Blood Diseases and Thrombosis Prevention and Treatment, The First People’s Hospital of Yunnan Province, Kunming University of Science and Technology Affiliated Hospital, Kunming, China; 2 Yunnan Province Clinical Research Center for Hematologic Disease, The First People’s Hospital of Yunnan Province, School of Medicine, Kunming University of Science and Technology, Kunming, China; 3 Department of Urology, The First People’s Hospital of Yunnan Province, Kunming University of Science and Technology Affiliated Hospital, Kunming, China; 4 State Key Laboratory of Primate Biomedical Research, Institute of Primate Translational Medicine, Kunming University of Science and Technology, Kunming, China; 5 Department of Integrated Traditional Chinese and Western Medicine, The First People’s Hospital of Yunnan Province, Kunming University of Science and Technology Affiliated Hospital, Kunming, China; 6 Department of Nephrology, Kumamoto University Graduate School of Medical Sciences, Kumamoto, Japan; 7 Department of Geriatrics, Peking University Shenzhen Hospital, Shenzhen, Guangdong, China

**Keywords:** Aprotinin, Cathepsin S (CTSS), CD4^+^ T cells, chronic kidney disease (CKD), CIBERSORT

## Abstract

**Background:**

Chronic kidney disease involves progressive fibrosis and immune dysregulation. Aprotinin, a broad-spectrum serine protease inhibitor, has shown dose-dependent renal effects, but its role in obstructive nephropathy is unclear.

**Methods:**

A unilateral ureteral obstruction (UUO) mouse model was used to assess aprotinin at 0.5 mg/day and 1 mg/day delivered via osmotic pumps for 7 days. Renal injury and fibrosis was evaluated by histology, serum markers, and expression of inflammation- and fibrosis-related genes. Mechanistic insights were obtained through LC-MS/MS proteomic profiling, functional enrichment analysis, and CIBERSORT-based immune deconvolution. Key proteins, signaling pathways, and immune cell infiltration were validated by Western blotting, immunohistochemistry, and flow cytometry.

**Results:**

Aprotinin at 1 mg/day significantly reduced tubular injury and interstitial fibrosis, whereas the 0.5 mg/day dose showed minimal benefit. The higher dose caused mild increases in serum creatinine and blood urea nitrogen. Proteomic analysis identified differentially expressed proteins enriched in immune regulatory pathways. Cathepsin S (CTSS), a protease involved in antigen presentation, was markedly decreased by aprotinin. CIBERSORT revealed reduced follicular helper T cells and partial restoration of naïve CD4^+^ T cells following treatment. These changes were further confirmed by flow cytometry. Western blotting and immunohistochemistry confirmed reduced CTSS and CD4 expression, along with decreased CD4^+^ T-cell infiltration and inhibition of ERK signaling.

**Conclusions:**

Aprotinin attenuates renal fibrosis in UUO in a dose-dependent manner. Its effects are associated with suppression of CTSS, inhibition of ERK signaling, and modulation of CD4^+^ T-cell subsets, and reduced immune cell infiltration. These findings indicate that aprotinin attenuates renal fibrosis through immunomodulatory mechanisms and support further investigation of serine protease inhibitors in immune-mediated kidney injury.

## Graphical Abstract

**Figure:**
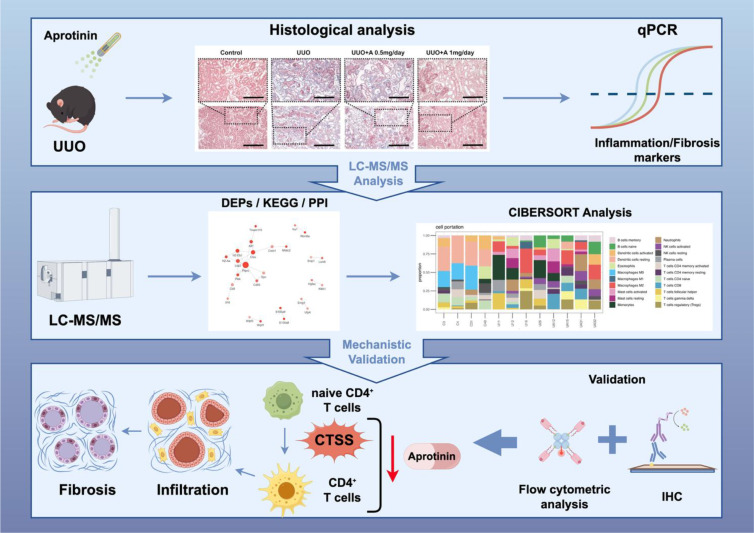
Aprotinin treatment in the unilateral ureteral obstruction (UUO) model mitigates renal injury and fibrosis, as demonstrated by histological analysis, qPCR, proteomic profiling, and CIBERSORT-based immune deconvolution. Mechanistic validation using immunohistochemistry, Western blotting, and flow cytometry further confirmed that aprotinin suppresses CTSS expression and reduces CD4^+^ T-cell infiltration, thereby contributing to the attenuation of renal inflammation and fibrosis. This figure was created by figdraw.com.

## Introduction

Chronic kidney disease (CKD) affects over 500 million people worldwide, with a global prevalence approaching 10%, and remains a significant public health and clinical challenge (1, 2). A hallmark of CKD is the progressive development of renal fibrosis and inflammation, which drive the gradual and irreversible decline in kidney function (3–5). This pathological process involves capillary rarefaction, tubule atrophy, excessive deposition of extracellular matrix components, activation of myofibroblasts, and infiltration of immune cells (6, 7). To investigate the molecular and cellular mechanisms underlying renal fibrosis, the unilateral ureteral obstruction (UUO) model is widely used in preclinical research, as it faithfully recapitulates the histopathological features of renal fibrosis and allows for controlled investigation of therapeutic interventions (8). Despite growing insights into CKD pathophysiology, effective therapies that directly target renal fibrosis remain lacking. Given the critical role of inflammation and immune dysregulation in fibrosis progression, modulating immune responses represents a promising therapeutic strategy.

Aprotinin is a broad-spectrum serine protease inhibitor first discovered in the 1930s. Clinically, it was used for decades to reduce blood loss during cardiac and other major surgeries due to its antithrombotic and anti-inflammatory properties (9). However, early clinical concerns about increased risks of acute kidney injury (AKI) and mortality led to its market withdrawal in 2007 (10, 11). Subsequent re-evaluation including a large meta-analysis of 88 trials involving over 15,000 patients found no significant increase in mortality compared to other antifibrinolytics (12). Based on this evidence, the European Medicines Agency (EMA) reapproved aprotinin in 2012 for restricted use in high-risk coronary artery bypass graft (CABG) surgery (13).

Beyond its hemostatic use, aprotinin has shown renoprotective effects in experimental models of acute kidney injury, particularly ischemia–reperfusion (I/R) injury. It has been reported to reduce tubular apoptosis, suppress proinflammatory mediators such as inducible nitric oxide synthase (iNOS), and downregulate signaling pathways including p38 MAPK and caspase-8 (14, 15). Additionally, aprotinin reduces thromboxane A2 (TxA2) production stimulated by kinins, thereby improving renal function in acute obstruction models (16). Importantly, the effects of aprotinin appear dose-dependent: high doses (e.g., 2 mg/day) can induce renal injury in healthy mice, while lower doses (e.g., 0.5–1 mg/day) may have protective effects depending on the disease context (17, 18).

Despite these findings, the effects of aprotinin in chronic models of obstructive nephropathy, such as UUO, have not been fully elucidated. In particular, the molecular mechanisms by which aprotinin may influence fibrosis through immune modulation remain unclear. Given the central role of protease–immune interactions in kidney injury and repair, it is essential to better understand how aprotinin affects immune cell dynamics and protease activity in the fibrotic kidney.

In this study, we employed a UUO mouse model to evaluate the dose-dependent effects of aprotinin on renal injury and fibrosis. Through proteomic profiling and immune cell deconvolution analysis, we identified key molecular pathways and immune cell subsets affected by aprotinin treatment. Our findings provide new insights into the immunomodulatory actions of serine protease inhibitors and highlight their potential as therapeutic agents for immune-driven kidney diseases.

## Materials and methods

### Animal studies

All animal procedures were approved by the Institutional Animal Care and Use Committee of Kunming University of Science and Technology (Approval No. K2023-0061) and conducted in accordance with the Guide for the Care and Use of Laboratory. Three-month-old male C57BL/6 mice were housed under standard conditions (22 ± 1 °C, 55 ± 2% humidity, 12-h light/dark cycle) with free access to food and water. Mice were randomly assigned to four groups: control (n = 4), UUO (n = 5), aprotinin 0.5 mg/day (n = 5), and aprotinin 1 mg/day (n = 5). Aprotinin (Sigma-Aldrich, USA) was dissolved in physiological saline using ultrasonic treatment to ensure complete solubility. Osmotic mini-pumps (model 1001W, RWD Life Science, Shenzhen, China) were implanted subcutaneously under isoflurane anesthesia to deliver either saline (control and UUO groups) or aprotinin (0.5 or 1 mg/day). Following pump implantation, UUO was induced by ligating the right ureter in the UUO and aprotinin groups, while control mice underwent sham surgery without ligation. After one week, blood samples were collected, and kidneys were harvested for further analysis. Kidneys were either snap-frozen in liquid nitrogen or fixed in 4% paraformaldehyde. Blood urea nitrogen (BUN) and serum creatinine levels were measured using a Cobas c311 automatic biochemical analyzer (Roche Diagnostics).

### Histological studies

Fixed kidneys were paraffin-embedded and sectioned at 3 μm thickness. Sections were stained with hematoxylin and eosin (H&E) or Masson’s trichrome to assess histopathological changes. The fibrotic area and tubular lumen area were quantified using ImageJ (National Institutes of Health, Bethesda, MD, USA). Tubular injury was scored semi-quantitatively as previously described, based on the percentage of tubules displaying necrosis, cast formation, dilation, or loss of the brush border, using a scale of 0–5: 0, 0%; 1, 1–10%; 2, 11–25%; 3, 26–45%; 4, 46–75%; 5, 76–100% (19). All histological assessments were performed in a blinded manner in five randomly selected fields per section at 200 × magnification.

### Protein extraction and LC-MS/MS analysis

Proteins were extracted from samples ground in liquid nitrogen and lysed in buffer containing 8 M urea (Sigma-Aldrich), 1% protease inhibitor (Merck Millipore), 3 μM trichostatin A (TSA, MedChemExpress), and 50 mM nicotinamide (NAM, Sigma-Aldrich). Lysates were sonicated and centrifuged at 12,000 × g for 10 min at 4 °C to remove debris. Protein concentration was determined using the bicinchoninic acid (BCA) assay (Beyotime Biotechnology). Equal amounts of protein were precipitated with pre-chilled acetone, washed 2–3 times, air-dried, and dissolved in 200 mM tetraethylammonium bromide (TEAB, Sigma-Aldrich). For protein digestion, samples were reduced with 5 mM DL-dithiothreitol (DTT, Sigma-Aldrich) at 56 °C for 30 min and alkylated with 11 mM iodoacetamide (IAM, Sigma-Aldrich) at room temperature for 15 min in the dark. Proteins were then digested overnight with trypsin (Promega) at an enzyme-to-protein ratio of 1:50 (w/w). Peptides were separated using an Easy-nLC 1000 system (ThermoFisher Scientific) with a gradient of 6%-90% acetonitrile (0.1% formic acid) at a flow rate of 500 nL/min. Mass spectrometry was performed on a timsTOF Pro instrument in data-independent acquisition parallel accumulation serial fragmentation (dia-PASEF) mode. The scan range was set to 300–1500 m/z, with 20 PASEF scans per cycle.

### Bioinformatics analysis

Differentially expressed proteins (DEPs) were annotated using the eggNOG-mapper tool based on the EggNOG database to extract Gene Ontology (GO) IDs, which were categorized into cellular components, molecular functions, and biological processes for functional annotation. KEGG pathway annotation was conducted by aligning the identified proteins against the KEGG database using BLAST (blastp, e-value ≤ 1e-4), with the highest-scoring alignment selected for annotation. Subcellular localization was predicted using the WolF PSORT software. Immune cell abundance was estimated with CIBERSORT by integrating protein expression data with the LM22 signature matrix, using a support vector regression algorithm to calculate the relative abundance of 22 immune cell subtypes. Protein sequences or database identifiers were queried in the STRING database to construct protein-protein interaction (PPI) networks, with high-confidence interactions (confidence score > 0.7) extracted. Visualization of PPI networks was performed using the R package “visNetwork.”

### Western blotting

Kidney tissues were lysed in ice-cold RIPA buffer (Thermo Scientific, USA) supplemented with protease inhibitors using a bead homogenizer. After centrifugation at 12,000 × g for 15 minutes at 4 °C, the supernatants were collected, and protein concentrations were determined using a BCA Protein Assay Kit (Thermo Scientific, USA). Equal amounts of protein were separated by 12% SDS-PAGE and transferred to PVDF membranes (Millipore, USA). Membranes were blocked with 5% non-fat milk in TBST for 1 hour at room temperature, followed by overnight incubation at 4 °C with primary antibodies against CTSS (Abclonal, A13482, China), CD4 (Abclonal, A26036PM, China), CD45 (Proteintech, 20103-1-AP, China), p-ERK (Proteintech, 28733-1-AP, China), ERK (Proteintech, 16443-1-AP, China), and GAPDH (Proteintech, 60004-1-Ig, China). After washing, membranes were incubated with HRP-conjugated secondary antibodies for 1 hour at room temperature. Signals were visualized using the ChemiScope imaging system (CLiN, China) with enhanced chemiluminescence. Band intensities were quantified using ImageJ software (NIH, USA) by an investigator blinded to the experimental groups.

### Immunohistochemistry

Kidney tissues were fixed in 4% paraformaldehyde, embedded in paraffin, and sectioned at a thickness of 3 µm. The sections were deparaffinized in xylene and rehydrated through a graded ethanol series. For antigen retrieval, they were incubated in citrate buffer (pH 6.0) at 95 °C for 30 minutes. Endogenous peroxidase activity was quenched by treatment with 3% hydrogen peroxide for 10 minutes. After blocking with 5% BSA, the sections were incubated overnight at 4 °C with primary antibodies against CD4 (ABclonal, A26036PM, China) and CTSS (ABclonal, A13482, China). Following washing, the VECTASTAIN^®^ Universal Elite ABC Kit (Vector Laboratories, PK-6200, USA) was applied according to the manufacturer’s instructions. Signals were visualized using a DAB detection system and counterstained with hematoxylin. Images were captured at 200× magnification using a Leica DM6 B Upright Microscope (Leica Microsystems, DM6B, Germany). For quantification, ten random non-overlapping fields were selected per mouse, and either the number of CD4^+^ cells or the percentage of CTSS-positive area was semi-quantitatively analyzed using ImageJ (National Institutes of Health, Bethesda, MD, USA) by a blinded investigator.

### Quantitative real-time PCR

Total RNA was extracted from kidney tissue using an RNA extraction kit (Promega, USA) and reverse-transcribed to cDNA with a commercial kit (Promega). Conventional and real-time PCR were conducted using a Mastercycler thermal cycler (Eppendorf, Germany) and StepOnePlus Real-Time PCR System (Applied Biosystems), respectively. Amplifications were performed using 2× Taq Universal PCR Master Mix (Promega). Primer sequences were designed using GeneScript software (GenScript Biotech Corp, China) and were as follows: Ccl2 (forward: TTAAAAACCTGGATCGGAACCAA, reverse: GCATTAGCTTCAGATTTACGGGT), Fas (forward: GCGGGTTCGTGAAACTGATAA, reverse: GCAAAATGGGCCTCCTTGATA), Fn1 (forward: GCTCAGCAAATCGTGCAGC, reverse: CTAGGTAGGTCCGTTCCCACT), Bax (forward: AGACAGGGGCCTTTTTGCTAC, reverse: AATTCGCCGGAGACACTCG), Col1a1 (forward: CTGGCGGTTCAGGTCCAAT, reverse: TCCAAACCACTGAAGCCTCG), Tnf-α (forward: GGTGATCGGTCCCCAAAGGGATGA, reverse: TGGTTTGCTACGACGTGGGCT), and Tgfb1 (forward: CGTGGAAATCAACGCTCCAC, reverse: ACTTCCAACCCAGGTCCTTC). Mouse Gapdh (forward: AAGAGGGATGCTGCCCTTAC, reverse: TACGGCCAAATCCGTTCACA) was used as the endogenous control, and gene expression fold changes were normalized to Gapdh expression.

### Isolation of kidney mononuclear cells

Mice were anesthetized and perfused with cold phosphate-buffered saline (PBS) to minimize contamination by circulating blood cells. Kidneys were rapidly harvested, decapsulated, and mechanically dissociated in RPMI-1640 medium (Gibco, C11875599BT, USA). The tissue was enzymatically digested in RPMI-1640 containing 0.5 mg/mL collagenase P (Roche, 11213857001, Switzerland) to facilitate cell dissociation.

The resulting suspension was filtered through a 70-μm cell strainer (Biosharp, BS-70-CS, China) to remove tissue debris. After centrifugation at 400 × g for 5 min at 4 °C, the cell pellet was resuspended in cold DPBS. Mononuclear cells were then enriched using Percoll density-gradient centrifugation. A discontinuous gradient was prepared by layering 36% Percoll over 72% Percoll in a 15-mL centrifuge tube. The cell suspension was carefully layered on top of the gradient and centrifuged at 400 × g for 30 min at room temperature. Cells collected from the interphase were washed twice with staining buffer and resuspended for subsequent flow cytometric analysis.

### Flow cytometric analysis

Isolated kidney mononuclear cells were first incubated with anti-mouse CD16/CD32 Fc receptor blocking antibody (BioLegend, 156604, USA) for 10 min at 4 °C to reduce nonspecific binding. Cells were then stained with fluorochrome-conjugated antibodies for surface marker detection at 4 °C for 25 min in the dark. The following antibodies were used: FITC anti-mouse CD4 (BioLegend, 100406, USA), APC anti-mouse CD185 (CXCR5) (BioLegend, 145505, USA), APC anti-mouse CD62L (BioLegend, 104411, USA), and PE anti-mouse/human CD44 (BioLegend, 103007, USA). After staining, cells were washed twice with FACS buffer and analyzed using a LSRFortessa flow cytometer (BD Biosciences, USA). Lymphocytes were first gated based on forward scatter (FSC) and side scatter (SSC) parameters to exclude debris and dead cells. At least 10,000 lymphocyte events were acquired for each sample. Data were analyzed using FlowJo software (FlowJo LLC, USA). The percentages of CD4^+^ T-cell subsets, including naïve CD4^+^ T cells (CD62L^+^ CD44^low^) and Tfh cells (CD4^+^ CXCR5^+^), were calculated within the gated lymphocyte or CD4^+^ T-cell populations as appropriate.

### Statistical analysis

Data are presented as mean ± standard deviation (SD). Statistical analyses were performed using GraphPad Prism software (version 9.5.1, GraphPad Software, San Diego, CA, USA). Comparisons among three or more groups were conducted using one-way analysis of variance (ANOVA) with Tukey’s test for normally distributed data or the Kruskal-Wallis test for non-parametric data. A p-value < 0.05 was considered statistically significant.

## Results

### Aprotinin alleviates renal pathology in the UUO model

**Figure 1:**
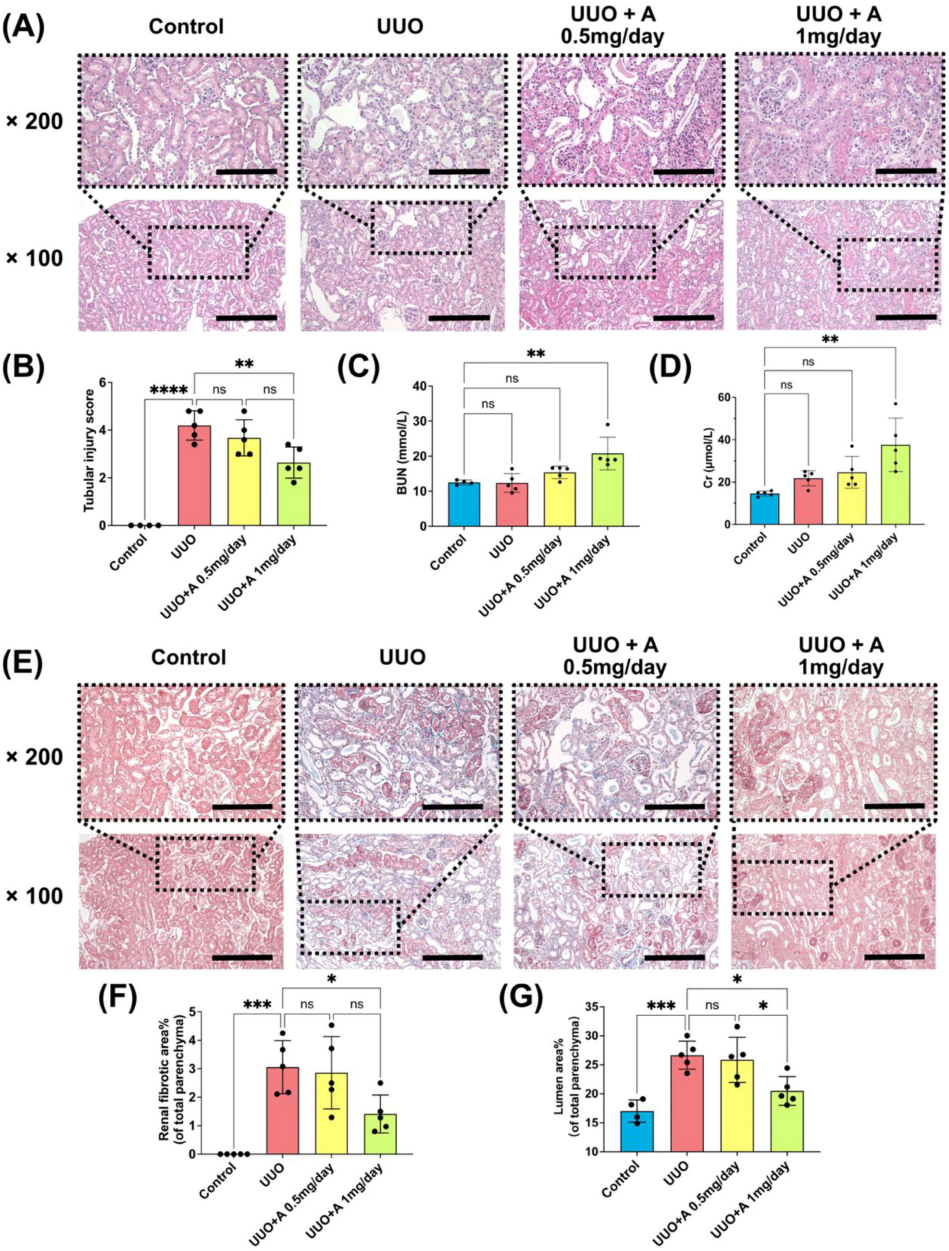
Effects of aprotinin on renal injury and fibrosis in the UUO model. **(A)** Representative images of H&E-stained kidney sections at 100× and 200× magnification (scale bar: 200 μm for 100×, 100 μm for 200×); **(B)** Tubular injury scores evaluated at 200× magnification based on a scale of 0 - 5 (n = 5 fields/slide); **(C, D)** Blood urea nitrogen (BUN) and serum creatinine (Cr) levels measured on day 7 post-surgery (n = 4 for the control group, n = 5 for UUO, UUO + A 0.5 mg/day, and UUO + A 1 mg/day groups each); **(E)** Representative images of Masson’s trichrome-stained mouse kidney sections at 100× and 200× magnification (scale bar: 200 μm for 100×, 100 μm for 200×); **(F, G)** Quantification of renal fibrotic area (%) and tubular lumen area (%) analyzed at 100× magnification. Data are presented as mean ± SD. Statistical significance was analyzed using one-way ANOVA. ns, not significant, *: p < 0.05, **: p < 0.01, ***: p < 0.001, ****: p < 0.0001.

In the 7-day UUO model, treatment with aprotinin at 1 mg/day significantly ameliorated tubular injury in the obstructed kidney, as shown by H&E staining. In contrast, the 0.5 mg/day dose had no significant therapeutic effect (**Figures 1A, B**). Interestingly, although the higher dose mitigated local injury, it led to elevated serum creatinine and BUN levels, suggesting potential adverse effects on the contralateral kidney. These markers remained unchanged with the 0.5 mg/day dose (**Figures 1C, D**). Masson’s trichrome staining further demonstrated that 1 mg/day aprotinin significantly reduced fibrotic area and tubular lumen dilatation in the obstructed kidney, whereas the lower dose showed no improvement (**Figures 1E–G**).

**Figure 2:**
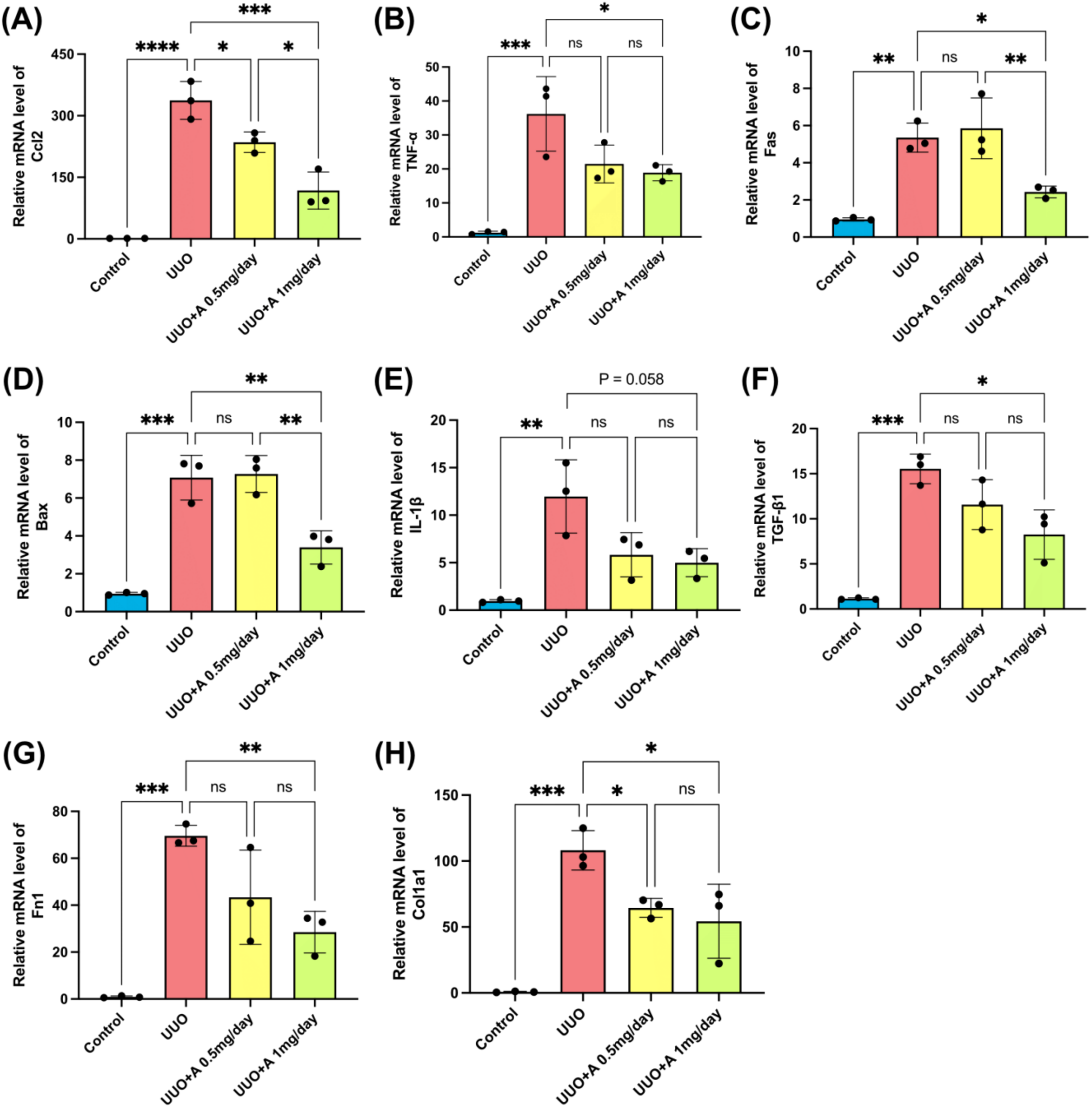
Effects of aprotinin on the expression of inflammation- and fibrosis-related genes in the UUO model. **(A–E)** Quantitative real-time PCR analysis of inflammatory markers, including Ccl2 **(A)**, TNF-α **(B)**, Fas **(C)**, Bax **(D)**, and IL-1β **(E)**; **(F–H)** Quantitative real-time PCR analysis of fibrosis-related markers, including TGF-β1 **(F)**, Fn1 **(G)**, and Col1a1 **(H)**. Data are presented as mean ± SD (n = 3 mice per group). Statistical significance was analyzed using one-way ANOVA. ns, not significant, *: p < 0.05, **: p < 0.01, ***: p < 0.001, ****: p < 0.0001.

At the molecular level, UUO markedly upregulated inflammatory genes (*Ccl2, TNF-α, Fas, Bax, IL-1β*) and fibrosis-associated genes (*TGF-β1, Fn1, Col1a1*). Aprotinin at 1 mg/day significantly suppressed the expression of all these factors, except *IL-1β*, which showed a non-significant reduction. The 0.5 mg/day dose only decreased *Ccl2* and *Col1a1* expression (**Figures 2A–H**).

### Proteomic profiling of differentially expressed proteins

**Figure 3:**
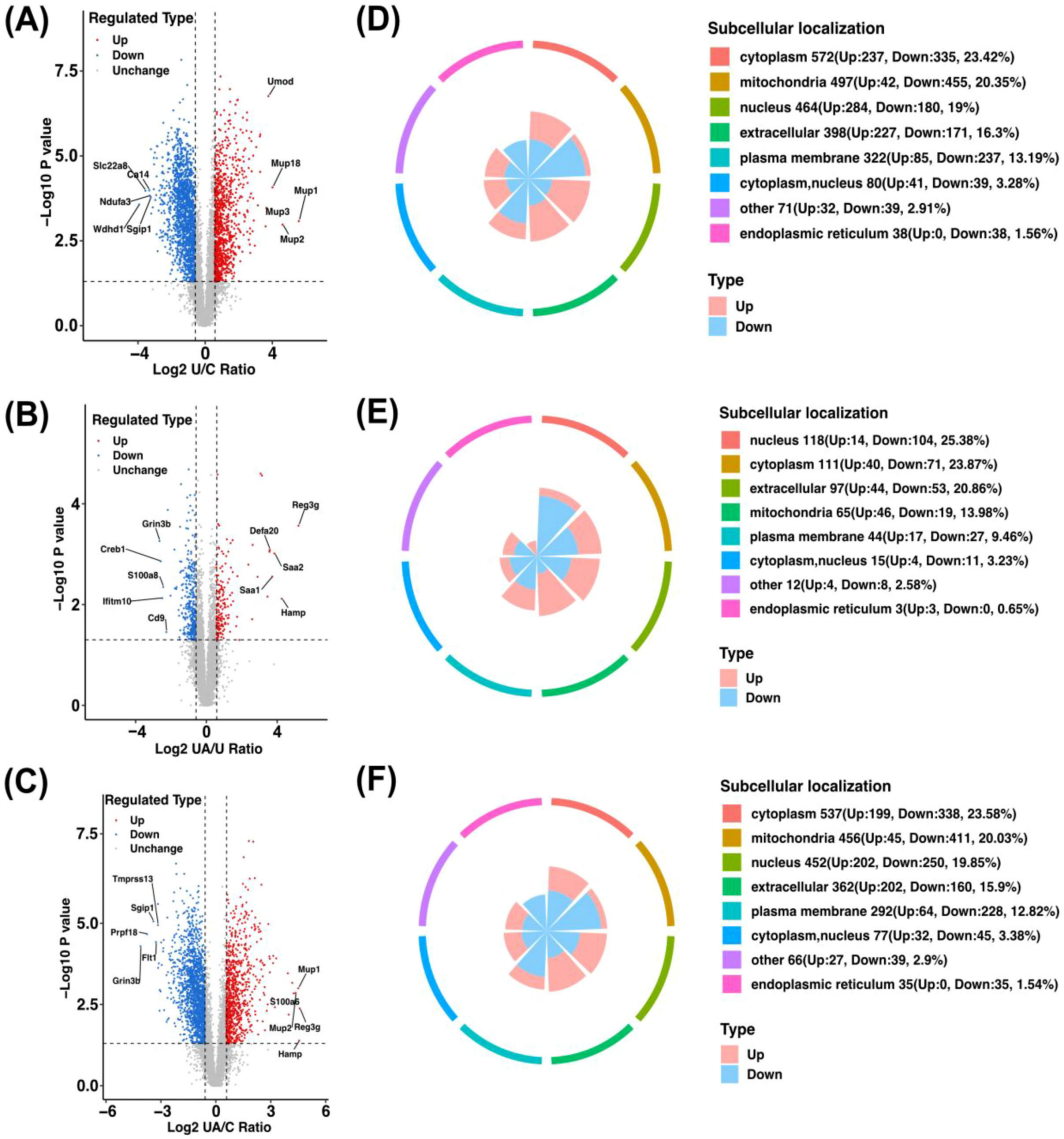
Proteomic analysis of DEPs in the UUO model treated with aprotinin (1 mg/day). **(A–C)** Volcano plots showing DEPs across experimental groups: **(A)** UUO vs. Control (U/C), **(B)** Aprotinin 1 mg/day vs. UUO (UA/U), and **(C)** Aprotinin 1 mg/day vs. Control (UA/C). Upregulated and downregulated proteins are highlighted in red and blue, respectively, with thresholds of fold change > 1.2 and p-value < 0.05; **(D–F)** Subcellular localization analysis of DEPs across experimental groups: **(D)** U/C, **(E)** UA/U, and **(F)** UA/C. Proteomic analysis was performed using kidney tissues from four mice per group (n = 4).

Label-free quantitative proteomics identified 6,571 proteins across control, UUO, and 1 mg/day aprotinin-treated groups. Based on a fold change >1.2 and p < 0.05, 2,442 DEPs (948 upregulated, 1,494 downregulated) were found between control and UUO groups; 465 DEPs (172 upregulated, 293 downregulated) between UUO and aprotinin groups; and 2,277 DEPs (771 upregulated, 1,506 downregulated) between control and aprotinin groups. The top five significantly altered proteins in each comparison were highlighted (**Figures 3A–C**). Subcellular localization analysis indicated that DEPs were mainly located in the cytoplasm, mitochondria, nucleus, extracellular space, and plasma membrane (**Figures 3D–F**).

### Function analysis of overlapping DEPs

**Figure 4:**
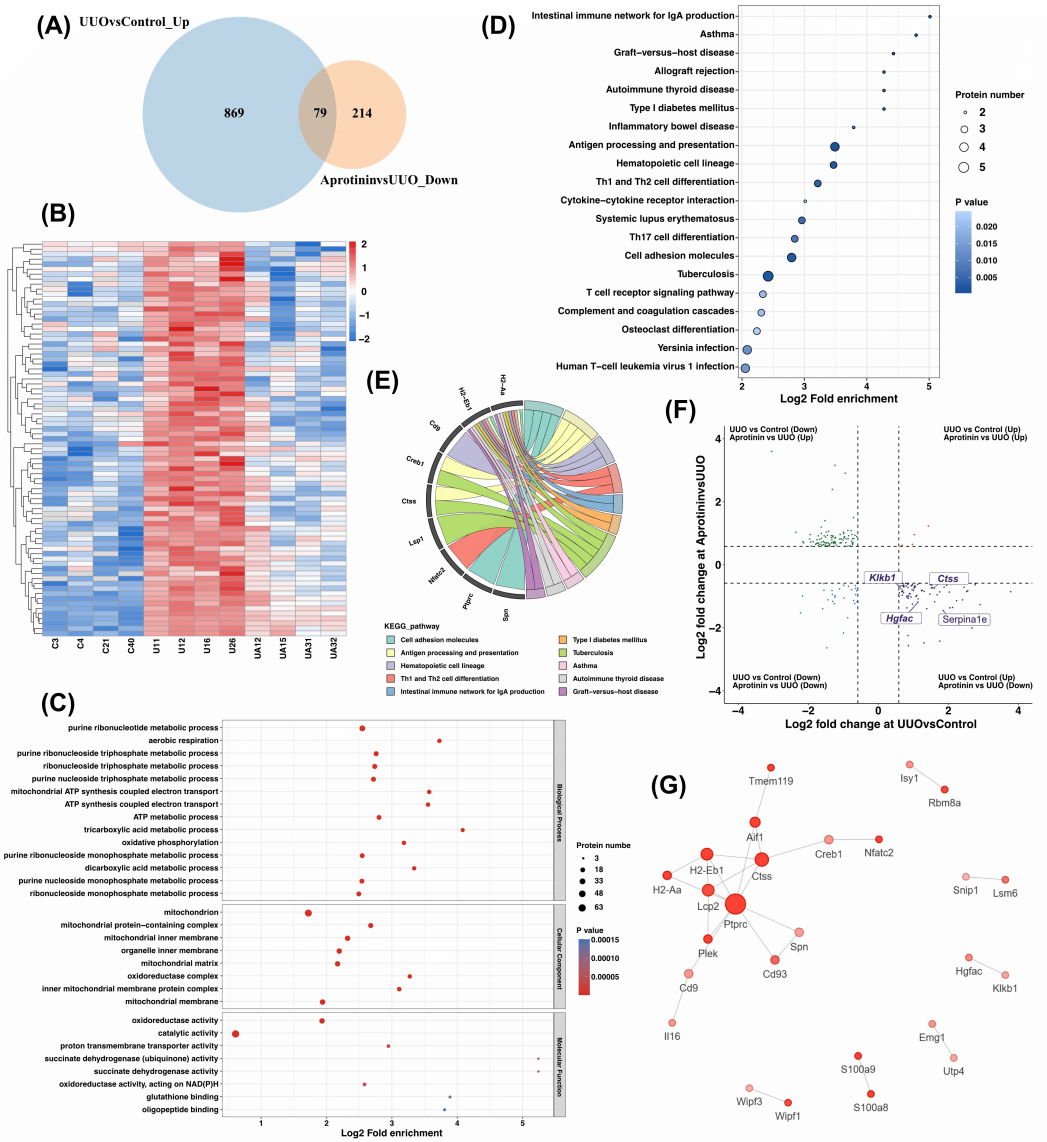
Functional analysis of overlapping DEPs upregulated in UUO vs. control and downregulated in aprotinin vs. UUO. **(A)** Overlap analysis of DEPs; **(B)** Hierarchical clustering of overlapping DEPs; **(C)** GO enrichment analysis; **(D)** KEGG pathway enrichment analysis; **(E)** Chord diagram linking overlapping DEPs to enriched pathways; **(F)** Nine-quadrant analysis of protease-related DEPs; **(G)** Protein-protein interaction (PPI) network. Proteomic analysis was performed using kidney tissues from four mice per group (n = 4).

To identify the potential targets of aprotinin, we intersected DEPs upregulated in UUO versus control with those downregulated in aprotinin versus UUO, revealing 79 overlapping proteins (**Figure 4A**). Hierarchical clustering showed distinct expression patterns across groups (**Figure 4B**). GO analysis indicated enrichment in neutrophil aggregation, kallikrein-kinin cascade, peptidyl-cysteine S-nitrosylation, MHC class II protein complex formation, and CD4 receptor binding (**Figure 4C**). KEGG analysis highlighted immune-related pathways such as antigen processing and presentation, Th1 and Th2 cell differentiation, Th17 cell differentiation, and cell adhesion molecules, though some were not directly relevant to renal fibrosis (**Figure 4D**). A chord diagram was constructed to visualize the associations between significantly enriched proteins and their respective pathways (**Figure 4E**).

Given aprotinin’s role as a serine protease inhibitor, we further analyzed protease-related DEPs, including Klkb1, Ctss, Hgfac, and Serpina1e (**Figure 4F**). Protein–protein interaction (PPI) analysis identified Cathepsin S (CTSS) as the most functionally connected protein, particularly in association with protein tyrosine phosphatase receptor type C (PTPRC, also known as CD45). Considering that CTSS contributes to antigen presentation and that CD45 is an essential molecule expressed on all mature T cells, their close linkage suggests a central role for CTSS in aprotinin-mediated immune modulation (**Figure 4G**).

To further investigate aprotinin’s mechanism, we analyzed DEPs downregulated in UUO vs. control and upregulated in aprotinin vs. UUO, identifying 96 overlapping proteins (**Supplementary Figure 1A, B**). GO analysis revealed enrichment in mitochondrial-related metabolic processes, particularly oxidative-reduction processes and energy metabolism, including the tricarboxylic acid (TCA) cycle and oxidative phosphorylation, emphasizing the potential involvement of mitochondrial function in the disease context (**Supplementary Figure 1C**). KEGG analysis confirmed involvement in in oxidative phosphorylation, amino acid metabolism, and glutathione metabolism, suggesting a role for mitochondrial regulation in disease progression and aprotinin’s effects (**Supplementary Figure 1D**). A chord diagram highlighted key links between DEPs and enriched metabolic pathways, with emphasis on the TCA cycle and oxidative stress response (**Supplementary Figure 1E**).

### Immune cell composition altered by aprotinin: insights from CIBERSORT

**Figure 5:**
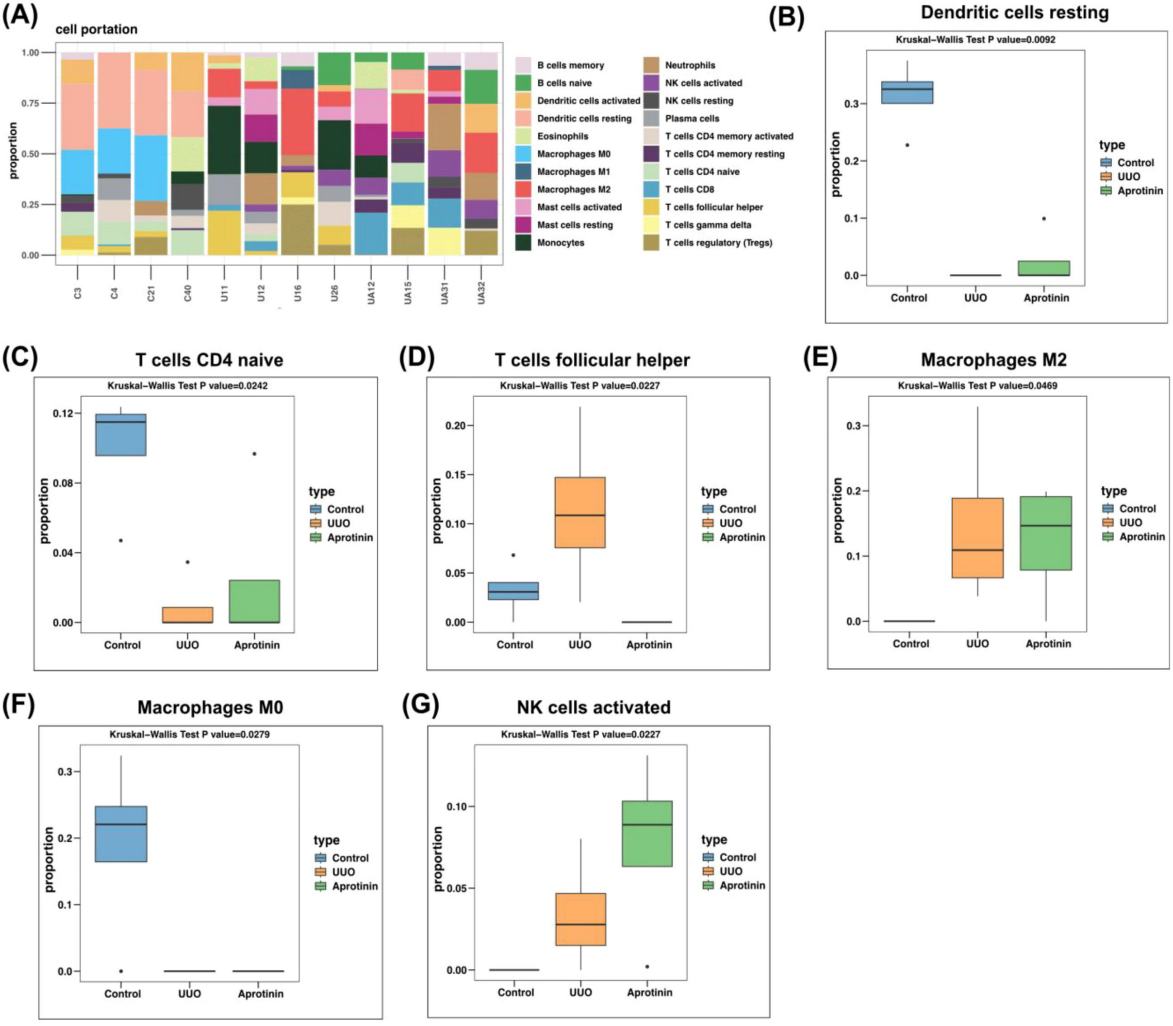
Immune cell composition in control, UUO, and aprotinin-treated (1 mg/day) groups. **(A)** Proportional distribution of 22 immune cell subtypes; **(B–G)** Proportions of selected immune cell types with significant differences: **(B)** Resting dendritic cells, **(C)** Naïve CD4 T cells, **(D)** Follicular helper T cells, **(E)** Macrophages M0, **(F)** Macrophages M2, **(G)** Activated NK cells. Data are presented as mean ± SD, and statistical significance was determined using the Kruskal-Wallis test. Proteomic analysis was performed using kidney tissues from four mice per group (n = 4).

Given the immunological associations of identified DEPs, CIBERSORT analysis was performed to assess immune cell composition across experimental groups. Significant changes were observed in multiple immune cell types (**Figure 5A**). Resting dendritic cells were decreased in the UUO group but partially restored with aprotinin (p = 0.0092) (**Figure 5B**). Naïve CD4^+^ T cells also declined in UUO but were partially recovered following treatment (p = 0.0242) (**Figure 5C**). In contrast, T follicular helper (Tfh) cells, a functional subset differentiated from CD4^+^ T helper cells, were significantly elevated in UUO but decreased after aprotinin treatment (p = 0.0227) (**Figure 5D**). Macrophage subsets exhibited modest alterations. M2 macrophages were increased in both the UUO and aprotinin-treated groups relative to controls, with no significant difference observed between the two groups, suggesting that macrophage polarization is unlikely to be the dominant mechanism underlying aprotinin-mediated protection (**Figure 5E**). Conversely, M0 macrophages were reduced in both UUO and aprotinin-treated kidneys compared with controls (**Figure 5F**). Activated NK cells were significantly elevated in UUO and further increased after aprotinin treatment (p = 0.0227) (**Figure 5G**). Other immune subsets, such as Tregs, activated mast cells, and neutrophils, showed variable changes, though these did not reach statistical significance.

### Aprotinin regulates CTSS, CD45, and CD4 expression and inhibits ERK signaling

**Figure 6:**
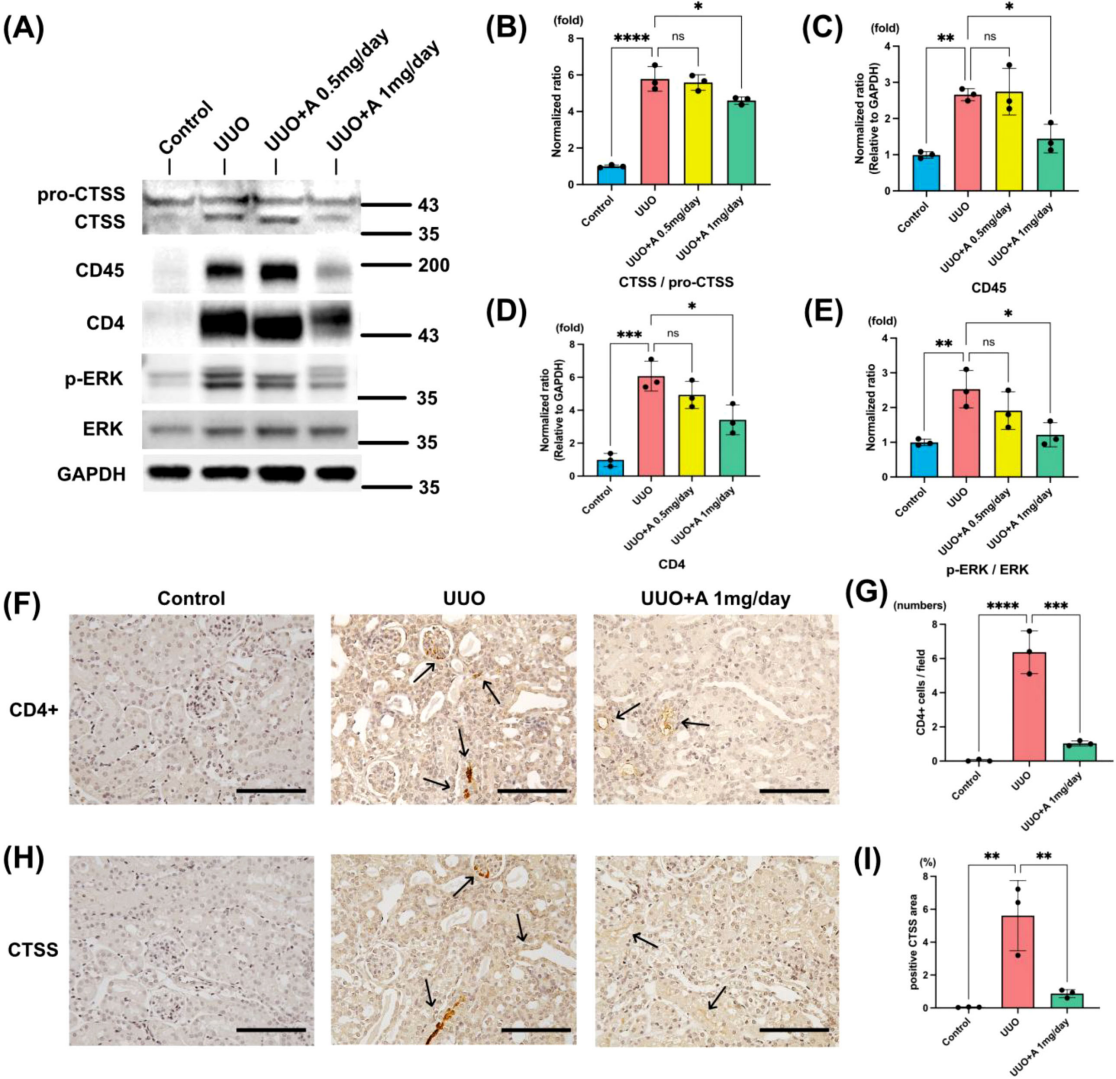
Aprotinin regulates CTSS, CD45, ERK phosphorylation, and CD4 expression in the UUO kidney. **(A)** Western blot analysis of CTSS, CD45, p-ERK, ERK, and CD4 expression in kidney tissues from Control, UUO, UUO + A 0.5 mg/day, and UUO + A 1 mg/day groups. GAPDH was used as the loading control. Molecular weight markers are indicated (kDa); **(B–E)** Densitometric analysis of the ratios of **(B)** CTSS/pro-CTSS, **(C)** CD45, **(D)** p-ERK/ERK, and **(E)** CD4; **(F, G)** Representative immunohistochemistry images of CD4^+^ cells **(F)** and quantification of CD4^+^ cells per field **(G)** in kidney sections; **(H, I)** Representative immunohistochemistry images of CTSS expression **(H)** and quantification of positive CTSS area (%) **(I)** in kidney sections. Photographed at 200× magnification (scale bar: 100 μm). Arrows indicate CD4^+^ cells and CTSS-positive areas. Data are presented as mean ± SD (n = 3 mice per group). Statistical significance was analyzed using one-way ANOVA. ns, not significant, *: p < 0.05, **: p < 0.01, ***: p < 0.001, ****: p < 0.0001.

Based on the preceding proteomic and CIBERSORT analyses which highlighted the roles of Tfh cells, we further investigated the effect of aprotinin on key related proteins and cell infiltration. UUO markedly increased the active form of CTSS (CTSS/pro-CTSS ratio), the expression of CD45, a characteristic surface molecule present on all mature T cells, the phosphorylation of ERK (p-ERK/ERK ratio), and CD4 protein levels. Treatment with 1 mg/day aprotinin significantly suppressed CTSS activation, reduced CD45 expression, inhibited ERK phosphorylation, and decreased CD4 protein levels (**Figures 6A–E**). In contrast, the 0.5 mg/day dose produced no significant changes in these molecular markers. Furthermore, immunohistochemical analysis provided visual and quantitative evidence that 1 mg/day aprotinin markedly reduced both CD4^+^ cell infiltration and the CTSS-positive staining area in UUO kidneys (**Figures 6F–I**).

### Aprotinin modulates CD4^+^ T cell subsets in the UUO kidney

**Figure 7:**
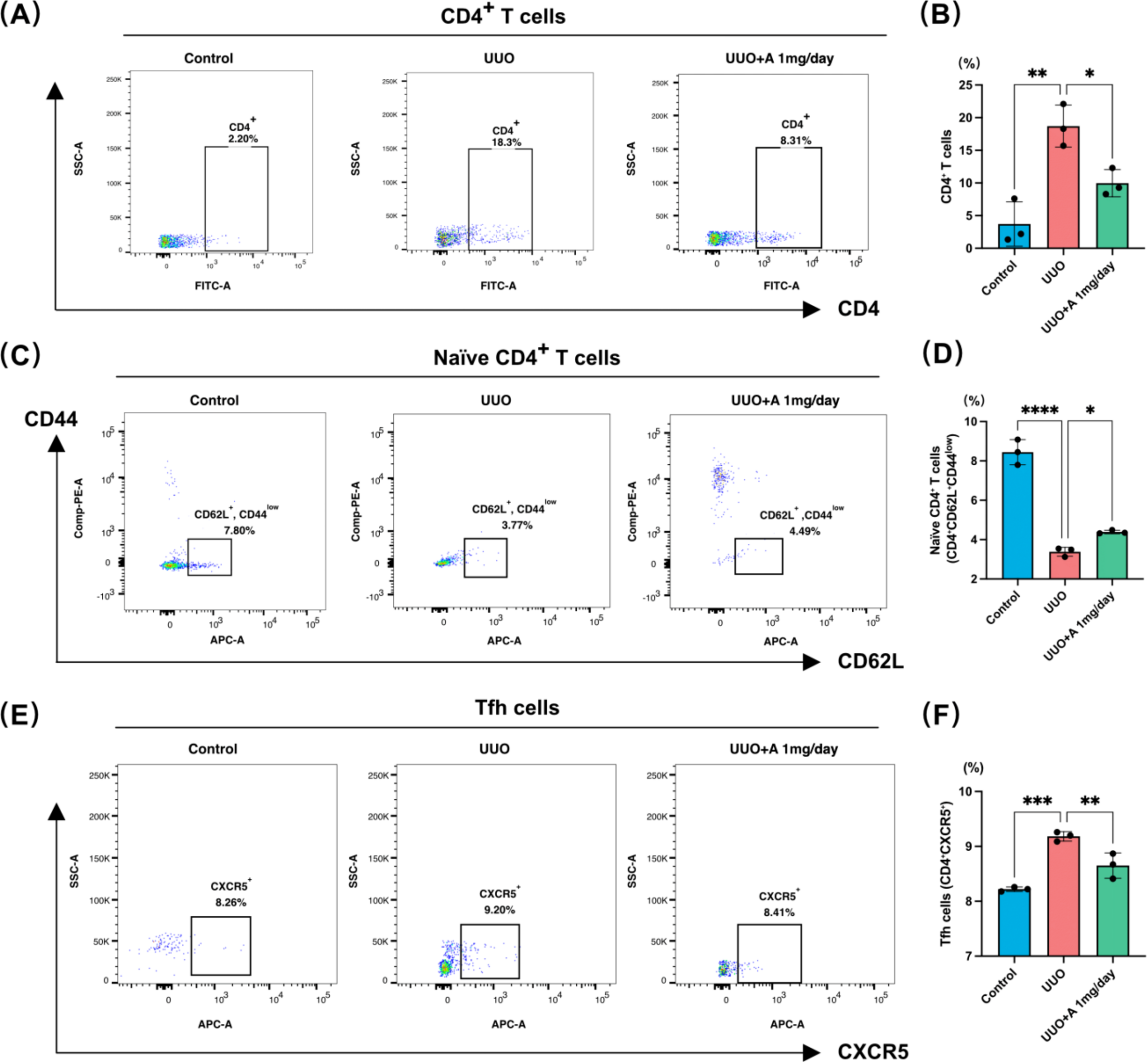
Aprotinin regulates CD4^+^ T-cell subsets in UUO-induced renal fibrosis. **(A)** Representative flow cytometry plots showing the proportion of CD4^+^ T cells among lymphocytes in Control, UUO, and UUO + A 1 mg/day groups; **(B)** Quantification of CD4^+^ T cells (%); **(C)** Representative flow cytometry plots of naïve CD4^+^ T cells (CD62L^+^ CD44^low^) among CD4^+^ T cells in the indicated groups; **(D)** Quantification of naïve CD4^+^ T cells (%); **(E)** Representative flow cytometry plots of Tfh cells (CD4^+^ CXCR5^+^) in the indicated groups; **(F)** Quantification of Tfh cells (%). Data are presented as mean ± SD (n = 3 mice per group). During lymphocyte isolation from kidney tissues, one sample in the Control group failed to yield sufficient mononuclear cells for flow cytometry analysis; therefore, the average value of the remaining two samples was used for statistical analysis. *: p < 0.05, **: p < 0.01, ***: p < 0.001, ****: p < 0.0001.

To further characterize the changes in CD4^+^ T-cell composition suggested by the preceding analyses, we analyzed CD4^+^ T cells and their major subsets by flow cytometry. The gating strategy used for identification of CD4^+^ T-cell subsets is shown in **Supplementary Figures 2A–E**. UUO markedly increased the proportion of CD4^+^ T cells among renal lymphocytes compared with the control group. Treatment with aprotinin at 1 mg/day significantly reduced the proportion of CD4^+^ T cells in UUO kidneys, indicating that aprotinin attenuates the accumulation of CD4^+^ T cells during renal injury (**Figures 7A, B****).** We next examined naïve CD4^+^ T cells defined as CD62L^+^ CD44^low^ cells. UUO markedly decreased the proportion of naïve CD4^+^ T cells compared with controls, suggesting a shift toward activated or differentiated T cell phenotypes during renal inflammation. Treatment with aprotinin partially restored the proportion of naïve CD4^+^ T cells in UUO kidneys, although the level remained lower than that of the control group (**Figures 7C, D**). Because previous analyses suggested a potential involvement of Tfh cells, we further evaluated the CXCR5^+^ CD4^+^ Tfh cell population. UUO significantly increased the proportion of Tfh cells relative to the control group, whereas treatment with aprotinin significantly reduced the abundance of this subset (**Figures 7E, F**). Collectively, these findings indicate that aprotinin not only reduces the overall infiltration of CD4^+^ T cells but also reshapes the composition of CD4^+^ T cell subsets in UUO kidneys, particularly by suppressing the expansion of Tfh cells.

## Discussion

This study demonstrated that aprotinin exerts dose-dependent protective effects against renal injury and fibrosis in a murine model of UUO. Treatment with 1 mg/day aprotinin significantly ameliorated tubular injury and interstitial fibrosis, as evidenced by histological improvements and decreased expression of key inflammatory and fibrotic markers. Interestingly, this dose was associated with mild elevations in BUN and serum creatinine, possibly reflecting hemodynamic changes in the contralateral kidney. Compensatory hyperperfusion in the non-obstructed kidney may potentially increase local drug exposure, enhancing sensitivity and contributing to subtle functional impairment. Proteomic analysis revealed extensive renal proteome remodeling following aprotinin treatment, identifying 79 overlapping DEPs enriched in immune regulatory pathways. Among these, CTSS, a key protease involved in antigen presentation and immune cell differentiation, was significantly downregulated. These findings suggest that aprotinin’s renoprotective effects may be mediated, in part, through modulation of immune responses and protease activity. In addition to immune-mediated mechanisms, renal fibrogenesis is also driven by resident kidney cells, including tubular epithelial cells, fibroblasts, and endothelial cells, which contribute to extracellular matrix deposition and tissue remodeling. Although the present study primarily focused on immune-related pathways, the potential effects of aprotinin on these resident renal cell populations warrant further investigation.

In this study, LC–MS/MS proteomic analysis identified CTSS and CD45 as key immune-related proteins associated with UUO-induced renal injury. Western blotting confirmed increased CD45 expression in UUO kidneys, indicating enhanced T-cell infiltration. Consistently, Western blotting and immunohistochemistry demonstrated elevated CD4 expression, which was suppressed by aprotinin treatment. CIBERSORT analysis suggested a redistribution of CD4^+^ T-cell subsets, characterized by reduced naïve CD4^+^ T cells and increased Tfh cells. These findings were further validated by flow cytometry, which confirmed that aprotinin reduced CD4^+^ T-cell and Tfh cell abundance while partially restoring naïve CD4^+^ T cells in UUO kidneys. CD4^+^ T cells are recognized as central mediators of chronic kidney injury, with Th2-polarized subsets shown to exacerbate fibrosis by promoting interstitial expansion, collagen deposition, and upregulation of profibrotic markers such as TGF-β, α-SMA, and fibronectin (20). Tfh cells, a specialized subset of CD4^+^ T cells, have emerged as key drivers of humoral immunity and are increasingly implicated in autoimmune kidney diseases such as systemic lupus erythematosus (SLE), membranous nephropathy (MN), and IgA nephropathy (IgAN), where their abundance correlates with autoantibody diversity, proteinuria, and renal dysfunction (21–24). Previous studies have demonstrated that CD4^+^ T cells are essential for the development of ischemic AKI, as CD4-deficient mice exhibit attenuated injury, while adoptive transfer of wild-type CD4^+^ T cells reinduce renal damage (25). In UUO, depletion of CD4^+^ T cells with monoclonal antibodies significantly alleviates fibrosis, whereas transfer of purified CD4^+^ (but not CD8^+^) T cells into RAG^-^/^-^ mice aggravates renal pathology (26). Moreover, in ischemia-reperfusion injury models, blockade of inducible T cell costimulator (ICOS) effectively suppresses Tfh activity and IL-21 production, leading to reduced fibrotic remodeling (27). In our study, UUO induced a marked reduction in naïve CD4^+^ T cells together with an expansion of Tfh cells, and these alterations were reversed by aprotinin, suggesting that aprotinin influences the immune microenvironment by modulating CD4^+^ T-cell dynamics, particularly affecting the Tfh compartment. However, the present study did not directly assess whether aprotinin modulates CD4^+^ T-cell differentiation *in vitro*, and further experiments using isolated CD4^+^ T cells will be required to determine whether aprotinin directly influences T-cell polarization.

Recent studies have strengthened the link between metabolic dysregulation and T cell-mediated fibrosis in UUO. In particular, succinate, a TCA cycle-derived metabolite, was shown to accumulate in obstructed kidneys and to promote renal infiltration and activation of CD4^+^ T cells by inducing CXCL9 and CXCL10 expression, ultimately driving myofibroblast activation through ERK signaling (28). This finding is consistent with our proteomic data showing enrichment of TCA cycle and oxidative phosphorylation pathways, as well as our observation that CD4^+^ T-cell expansion and ERK activation are attenuated by aprotinin.

CTSS is a cysteine protease responsible for processing the invariant chain and antigenic peptides in the MHC class II pathway (29, 30). Through its role in antigen processing, CTSS facilitates the expansion and T cell receptor diversification of Tfh cells, thereby promoting humoral immunity and contributing to disease progression in IgAN and SLE (31–33). Consistent with these immunological functions, our PPI analysis suggested that aprotinin may indirectly regulate immune responses through modulation of CTSS-associated pathways. Integrated bioinformatics analysis in essential thrombocythemia has likewise identified CTSS and PTPRC as central immune-related hub genes within PPI networks, further highlighting the immunoregulatory significance of CTSS (34). Based on these findings, we hypothesized that CTSS may serve as a critical mediator of aprotinin-induced immunomodulatory effects during renal injury. In addition to its role in antigen presentation, CTSS has been implicated in the regulation of immune activation and inflammatory signaling cascades. Previous studies have suggested that CTSS-mediated immune activation may engage downstream MAPK pathways, including ERK (35, 36). In the present study, aprotinin treatment markedly reduced ERK phosphorylation in UUO kidneys, indicating that attenuation of CTSS-associated immune signaling may be linked to the reduced immune cell infiltration and fibrotic responses observed after aprotinin treatment. Although aprotinin has classically been described as a broad-spectrum serine protease inhibitor targeting enzymes such as trypsin, plasmin, and kallikrein, recombinant variant studies have demonstrated that it can also inhibit Cathepsin G through specific residue substitutions (37). Moreover, cysteine proteases such as Cathepsin C have been shown to activate serine proteases like granzymes, indicating functional crosstalk between the two classes (38). Interestingly, in partial-obstruction hydronephrosis models characterized predominantly by mechanical stress rather than inflammation, CTSS has been reported to function mainly in extracellular matrix (ECM) remodeling and tissue repair, such that its inhibition may disturb matrix homeostasis and worsen injury (39). By contrast, in the acute, inflammation-driven UUO model used in our study, CTSS is more likely to accelerate fibrotic progression by enhancing macrophage recruitment and antigen presentation, thereby amplifying immune activation. Future studies employing cell type-specific genetic manipulation will be essential to delineate the context-dependent roles of CTSS in renal fibrosis.

Although 1 mg/day aprotinin has been reported to be safe in nephrotic syndrome model (17), we observed mild elevations in BUN and serum creatinine under UUO conditions. Notably, previous studies have shown that 2 mg/day aprotinin induces renal injury in healthy mice, whereas 0.5–1 mg/day does not cause overt toxicity (18). This may reflect altered drug distribution or enhanced sensitivity of the contralateral kidney due to hemodynamic remodeling. Clinical perfusion studies have shown that with increasing obstruction severity, the blood flow, blood volume, and clearance in the obstructed kidney decline significantly, whereas the contralateral kidney undergoes compensatory hyperperfusion (40). Likewise, dynamic 99mTc-MAG3 imaging in UUO mice demonstrated reduced uptake, delayed peak activity, and impaired excretion in the obstructed kidney, while contralateral function remained stable (41). These results suggest that pathological remodeling under UUO may alter aprotinin distribution and renal pharmacodynamics, even at doses considered safe in other contexts. However, it should be noted that the present study did not directly measure the pharmacokinetic profile of aprotinin in the UUO setting. Therefore, the observed functional changes may reflect altered drug exposure or sensitivity in the contralateral kidney secondary to hemodynamic remodeling, rather than intrinsic toxicity. Future studies incorporating pharmacokinetic measurements will be required to clarify the distribution and clearance of aprotinin under obstructive conditions. In contrast, we previously found that Camostat, a clinically used serine protease inhibitor, exerts robust therapeutic effects across multiple kidney injury models, including UUO, without any detectable toxicity (42–48). These findings highlight potential differences in the pharmacokinetic and immunological profiles of distinct protease inhibitors under disease-specific conditions and warrant further investigation into their safety windows and target specificity.

## Limitations

Several limitations should be acknowledged. First, although aprotinin demonstrated clear antifibrotic effects in the UUO model, the precise cellular targets and downstream signaling mechanisms remain incompletely defined. Second, although immune alterations were initially inferred from computational deconvolution of bulk proteomic data and partially validated by flow cytometry for CD4^+^ T cells and their subsets, the broader immune landscape still lacks comprehensive cell type–specific validation. Third, although CTSS emerged as a potential key regulator linking protease activity and immune modulation, its causal role was not directly validated through genetic or pharmacological intervention. Fourth, while previous studies have highlighted the importance of metabolic pathways such as the tricarboxylic acid (TCA) cycle in T-cell activation during obstructive nephropathy, our study did not directly quantify metabolic intermediates, and further metabolomic analyses will be required to clarify their contribution to aprotinin’s effects. Finally, only male mice were included in this study. Given that sex-related differences may influence immune responses and the progression of renal fibrosis, future studies incorporating both sexes will be necessary to confirm the generalizability of these findings.

## Conclusions

This study demonstrates that aprotinin attenuates renal fibrosis in the UUO model in a dose-dependent manner, with 1 mg/day but not 0.5 mg/day conferring significant protection. Integrated proteomic, CIBERSORT, and experimental validation analyses revealed that aprotinin suppresses CTSS activation, reduces CD4^+^ T-cell infiltration, and inhibits ERK signaling in UUO kidneys. Flow cytometric analysis further showed that aprotinin reshapes CD4^+^ T-cell subsets, characterized by decreased Tfh cells and partial restoration of naïve CD4^+^ T cells. These findings suggest that modulation of CTSS-associated immune pathways may contribute to the antifibrotic effects of aprotinin and highlight CTSS as a potential therapeutic target in immune-mediated kidney injury.

## Data Availability

The datasets presented in this study can be found in online repositories. The names of the repository/repositories and accession number(s) can be found below: PXD063655 (ProteomeXchange; https://proteomecentral.proteomexchange.org/?pxid=PXD063655).

## Supplementary material

Supplementary Figure 1 Functional analysis of overlapping DEPs downregulated in UUO vs. control and upregulated in aprotinin vs. UUO. **(A)** Overlap analysis of DEPs; **(B)** Hierarchical clustering of overlapping DEPs; **(C)** GO enrichment analysis; **(D)** KEGG pathway enrichment analysis; **(E)** Chord diagram linking overlapping DEPs to enriched pathways. Proteomic analysis was performed using kidney tissues from four mice per group (n = 4).

Supplementary Figure 2 Flow cytometry gating strategy. **(A)** Kidney mononuclear cells were first gated based on FSC-A and SSC-A to identify lymphocytes. **(B)** Doublets were excluded by gating on single cells. **(C)** CD4^+^ T cells were then selected. T follicular helper (Tfh) cells were identified as CXCR5^+^ cells **(D)**, and naïve CD4^+^ T cells were defined as CD62L^+^CD44^low^ cells **(E)**.
