# Supplementary material for: Proteomic insights into aprotinin’s immunomodulatory effects in attenuating renal fibrosis in a unilateral ureteral obstruction model
Source: Front Immunol. 2026 May 1;17:1751652. doi: 10.3389/fimmu.2026.1751652 (PMC13175798; doi:10.3389/fimmu.2026.1751652)
Supplement: Supplementary Figure 1 — Functional analysis of overlapping DEPs downregulated in UUO vs. control and upregulated in aprotinin vs. UUO. (A) Overlap analysis of DEPs; (B) Hierarchical clustering of overlapping DEPs; (C) GO enrichment analysis; (D) KEGG pathway enrichment analysis; (E) Chord diagram linking overlapping DEPs to enriched pathways. Proteomic analysis was performed using kidney tissues from four mice per group (n = 4). [file Supplementaryfile1.docx]

**Supplementary Figure 1. Functional analysis of overlapping DEPs downregulated in UUO vs. Control and upregulated in Aprotinin vs. UUO.**

**
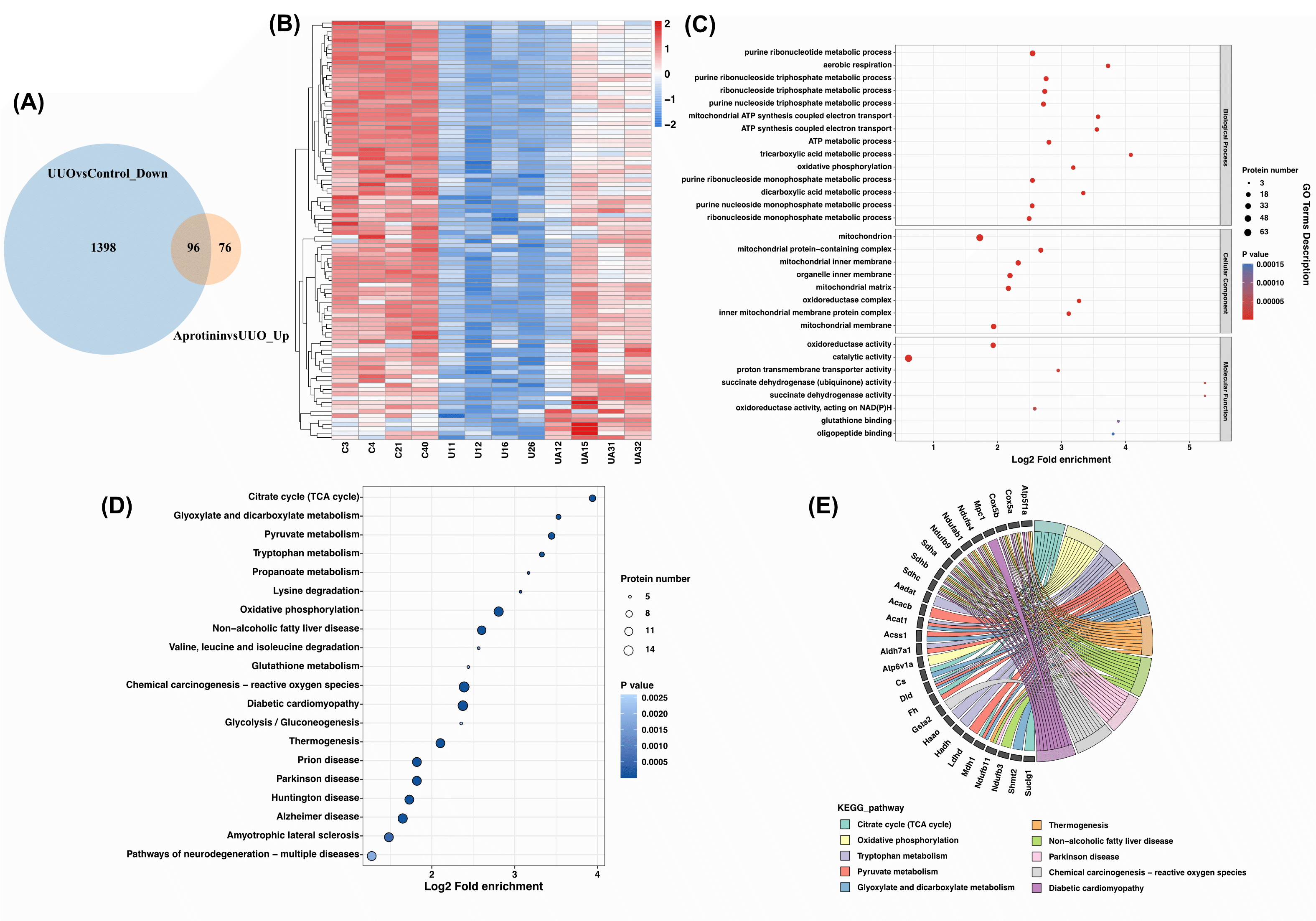
**

**(A)** Overlap analysis of DEPs; **(B)** Hierarchical clustering of overlapping DEPs; **(C)** GO enrichment analysis; **(D)** KEGG pathway enrichment analysis; **(E)** Chord diagram linking overlapping DEPs to enriched pathways. Proteomic analysis was performed using kidney tissues from four mice per group (n = 4).

**Supplementary Figure S2. Flow cytometry gating strategy.**


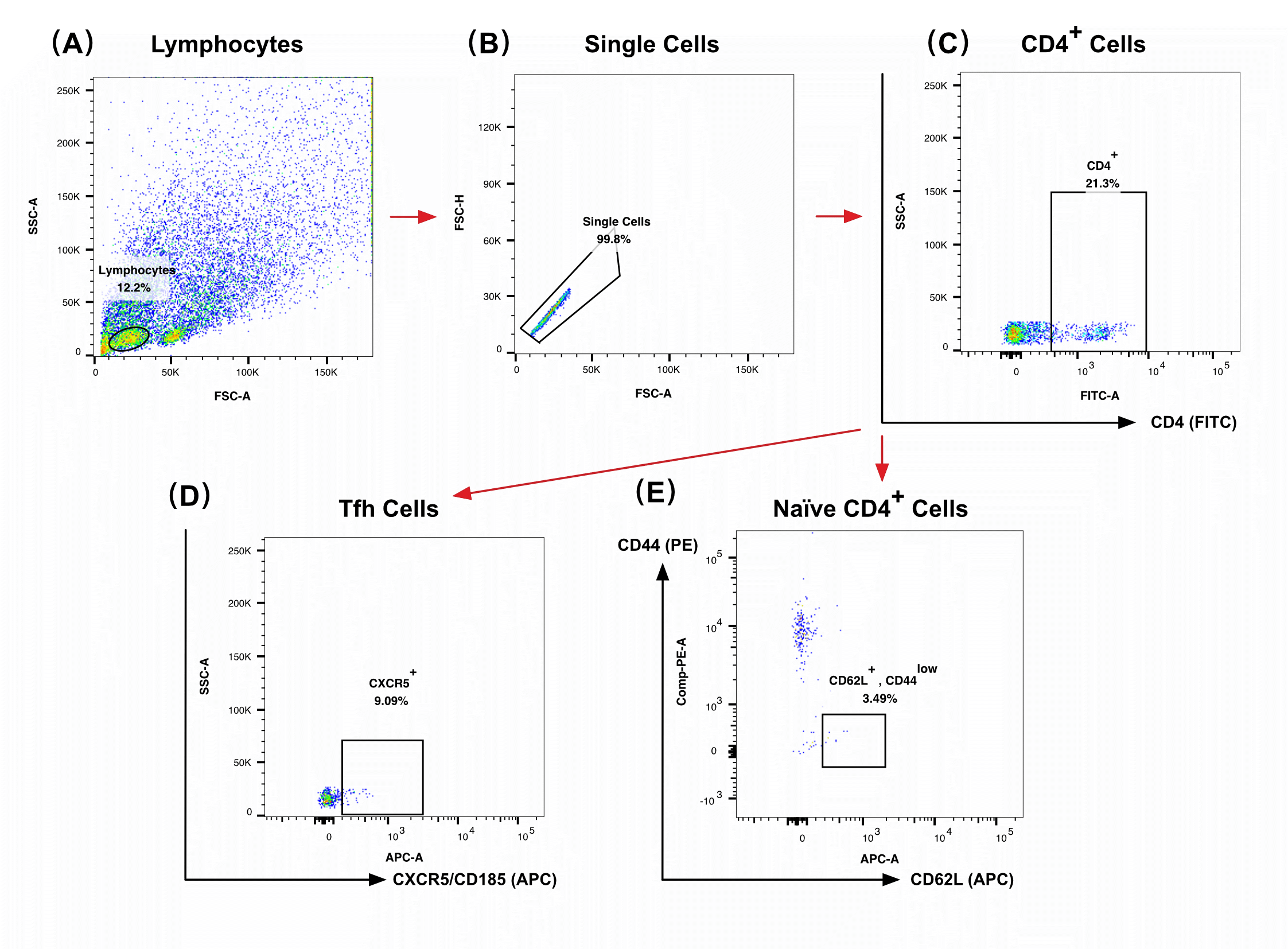


**(A)** Kidney mononuclear cells were first gated based on FSC-A and SSC-A to identify lymphocytes. **(B)** Doublets were excluded by gating on single cells. **(C)** CD4⁺ T cells were then selected. T follicular helper (Tfh) cells were identified as CXCR5⁺ cells **(D)**, and naïve CD4⁺ T cells were defined as CD62L⁺CD44^low^ cells **(E)**.
